# Clinical management of the most common extra-intestinal manifestations in patients with inflammatory bowel disease focused on the joints, skin and eyes

**DOI:** 10.1177/2050640620958902

**Published:** 2020-09-14

**Authors:** Fenna M Jansen, Stephan R Vavricka, Alfons A den Broeder, Elke MGJ de Jong, Frank Hoentjen, Willemijn A van Dop

**Affiliations:** 1 Department of Medicine, Division of Gastroenterology and Hepatology, Radboud University Medical Center, Nijmegen, The Netherlands; 2 Department of Medicine, Division of Gastroenterology and Hepatology, University Hospital Zurich, Zurich, Switzerland; ³Department of Medicine, Division of Rheumatology, Sint Maartenskliniek, Nijmegen, The Netherlands; 4 Department of Medicine, Division of Dermatology, Radboud University Medical Centre, Nijmegen, The Netherlands

**Keywords:** Inflammatory bowel disease, Crohn’s disease, ulcerative colitis, extra-intestinal manifestation, ocular manifestation, musculoskeletal manifestation, cutaneous manifestation, treatment, therapy

## Abstract

Extra-intestinal manifestations (EIMs) of inflammatory bowel disease (IBD) occur frequently and contribute to morbidity and reduced quality of life. The musculoskeletal, ocular and cutaneous organ systems are frequently involved in IBD-related EIMs. By focusing on manifestations involving the joints, skin and eyes, this review will discuss the most common clinically relevant and burdensome EIMs that affect IBD patients, and strives for early recognition, adequate treatment and timely referral. For this purpose, we aimed to create a comprehensive overview on this topic, with the main focus on the treatment of reactive and associated EIMs, including spondyloarthropathies, pyoderma gangrenosum, erythema nodosum, psoriasis and anterior uveitis. The recently developed biologicals enable simultaneous treatment of inflammatory disorders. This review can be used as a helpful guide in daily clinical practice for physicians who are involved in the treatment of IBD patients.

## Clinical case

A 45-year-old woman with a history of ulcerative colitis (UC) since 1999 presents with complaints of bloody diarrhoea and pain in her left-lower abdomen. Her dose of oral mesalamine is increased, and topical mesalamine is started. Concomitantly, she complains about new-onset joint pain in both ankles and her right wrist. During physical examination, one light-red bruise on her right shin is visible, which she explained as an injury due to a bicycle accident.

## Introduction

Inflammatory bowel diseases (IBD) consist of Crohn’s disease (CD) and ulcerative colitis (UC). They are characterised by a chronic relapsing and remitting disease course that results in intestinal symptoms but also frequently in extra-intestinal manifestations (EIMs).^1^,^2^ The latter can contribute to morbidity in IBD patients and can significantly reduce quality of life (QoL).^3^,^4^

The term ‘EIM’ covers all IBD-associated clinical manifestations located outside of the gastrointestinal tract.^1^,^2^ Based on the underlying pathophysiological and immunological mechanisms, EIM can be categorised into different but sometimes overlapping groups^1^ (Table 1). The most common group – and main topic of this review – is that of reactive manifestations. This group most likely shares pathophysiology with IBD but has different histological appearances. Reactive manifestations may follow the intestinal disease course or become manifest independent of intestinal disease.^1^,^5^,^6^ Most frequently involved are the musculoskeletal, cutaneous and ocular organ systems. Extra-intestinal disease complications are directly related to the intestinal disease activity or to disease-specific treatment, and include, for example, osteoporosis, malnutrition, kidney stones, gallstones and IBD drug-related symptoms.^1^,^2^,^5–9^ Associated diseases are less directly related to IBD compared to reactive EIMs, but are more distinctive diseases that are frequently observed in the IBD population and that might share similar genetic and immune-mediated pathways.^1^,^6^ Examples include axial spondylarthritis (SpA), including its archetype ankylosing spondylitis (AS), also known as Bechterew’s disease, or radiographic axial spondyloarthritis, primary sclerosing cholangitis (PSC), psoriasis and hidradenitis suppurativa (HS).^1^ A closely related or specific IBD manifestation, also called metastatic CD, shares the same histopathology as IBD and can be distinguished as a mucocutaneous form of CD specified as non-necrotising granulomas at other sites than the gastrointestinal tract.^9^,^10^ Urogenital (vulvar) and non-genital involvement (cutaneous/nasal ulcerations, nodules or plaques) are examples of this rare manifestation.^1^,^8^,^10^

**Table 1.** Overview of the different types of EIMs in IBD patients.

Type of EIM in IBD	Definition	Musculoskeletal	Cutaneous	Ocular
Reactive	Shared pathophysiology with IBD, but different histology	Axial SpAPeripheral SpA Non-inflammatory joint complaints	Erythema nodosumPyoderma gangrenosum	EpiscleritisScleritisUveitis
Complications	Consequences of intestinal disease activity or induced by IBD treatment	Drug-induced arthralgia (corticosteroid withdrawal, vedolizumab) Metabolic bone disease (osteoporosis)	Drug-induced skin manifestations, i.e. psoriasiform lesions (anti-TNF) e.g. drug hypersensitivity lesions (thiopurines)	Drug-induced glaucomaDrug-induced cataract (corticosteroids)
Associated	Might share genetic and immune-mediated pathways with IBD but are seen as distinctive entities	Axial SpA	Hidradenitis suppurativaPsoriasis	
Specific/closely related to IBD	Similar histopathology as IBD		Mucocutaneous/metastatic CD (e.g. genital or non-genital ulcerations/nodules)	

IBD: inflammatory bowel disease; EIM: extra-intestinal manifestations; SpA: spondylarthritis; CD: Crohn’s disease; TNF: tumour necrosis factor.

In general, 30–50% of IBD patients experience at least one EIM,^5^,^7^,^11–15^ with an overall higher incidence in CD,^5^,^7^,^11^,^13^,^16^,^17^ females,^7^,^13^,^16^,^17^ smokers^7^,^16^,^18^,^19^ and prolonged disease duration.^17^,^19^ The presence of one EIM comes with a higher probability for developing other EIMs.^5^,^11^,^16^,^20^ The majority of EIMs manifest after establishing a diagnosis of IBD, whereas uveitis and peripheral and axial arthritis precede the IBD diagnosis in 50%, 20% and 40% of patients, respectively.^7^,^11^,^16^

The presence of EIMs – and of reactive EIMs in particular – is still underreported by gastroenterologists due to a lack of awareness, time or diagnostic hurdles, and sometimes over-reported by patients. IBD patients themselves are also often not aware of the possible relation between their extra-intestinal complaints and their bowel disease, and so they may not report these complaints to their treating physician. In this narrative review, we will answer clinically relevant questions about the most commonly observed EIMs in order to broaden current knowledge about the occurrence, clinical picture, management and referral strategies.

## Method

We performed a PubMed search with the following MESH, Majr and tiab terms: inflammatory bowel, Crohn, Chron’s, ulcerative colitis, pyoderma gangrenosum (PG), erythema nodosum (EN), episcleritis, uveitis anterior, scleritis, spondyloarthritis, spondyloarthropathies, ankylosing spondylitis, sacroiliitis, arthralgia, spondylitis ankylosing, extraintestinal manifestation, eye diseases, ocular manifestation, ophthalmic manifestation, eye manifestation, skin diseases, skin manifestation, dermatological manifestation, dermatologic manifestation, arthralgia, joint, back pain.

The total search was limited to publication dates within the last 10 years at the time of the search (January 2020). The search revealed 1184 publications. We excluded 573 papers for reasons including unavailable in English language, less useful type of research (case-control studies), limited relevance of research questions or main outcomes (other diseases, not IBD-related) and other study population (children). After removing 68 duplicates, 315 (27.2%) articles were identified that met our inclusion criteria. The 228 articles left were scored as ‘maybe-useful’ while having other research questions of interests but could be used to ascertain relevant references. Using the snowball method, we found the original and more recently published papers useful for this review.

## Clinical case continued 1

Following the start of IBD therapy, the patient complains of less severe but long-term lower-back pain and morning stiffness for at least an hour but which improves by movement and exercise. In addition, stiffness in both wrists and fingers does not allow for daily activities such as opening jars and holding cups. There is no visible swelling or redness. What is the differential diagnosis here, and what treatment options are available?

## Musculoskeletal manifestations

What are the different types of arthropathies associated with IBD?

Arthropathies, an over-arching term for all types of joint disorders, can be classified according to the predominant localisation of symptoms (axial or peripheral) and according to the presence or absence of clinical joint inflammation, called inflammatory arthritis.^21–23^ Moreover, it is important to place arthropathies in the context of active or quiescent IBD, as this determinates the (treatment) approach.^24^ Of note, in Anglo-Saxon countries, the term ‘arthritis’ is also often used to cover non-inflammatory joint issues, hence the redundant term ‘inflammatory arthritis’. The term ‘clinical’ is important, as in the absence of clinical features of inflammation and in non-inflammatory arthritis such as osteoarthritis (term for degenerative joint disorder), subclinical inflammation can sometimes be demonstrated using imaging (ultrasound (US), radiographic imaging and magnetic resonance imaging) without having clinically relevant consequences.^21^,^25^,^26^

Inflammatory arthritis usually presents with signs of host response, including synovial swelling, thickening and/or hydrops, with symptoms such as pain, stiffness, warmth and sometimes redness.^21^ Inflammatory joint complaints (also known as clinically suspect arthralgia) are defined as patient-reported joint pain during the previous year, with stiffness of an hour or longer in the morning or after rest, improving upon on exercise, without the presence of arthritis yet.^21–23^ More specific criteria exist if for example rheumatoid arthritis has been suspected as an underlying disease for clinically suspected arthralgia.^27^

Arthritis can occur axially, mainly in sacroiliac (SI) joints and/or facet joints of the vertebrae. Arthritis in these joints leads to alternating buttock pain (sacroiliitis) and inflammatory back pain (SpA). Inflammatory back pain has been operationalised as morning stiffness, improvement upon exercise, no improvement upon rest and pain at night expressed before 45 years of age (being aware that immunosuppressives suppress and thereby postpone inflammatory symptoms).^2^,^21^,^26^,^28^,^29^ Arthritis in peripheral joints can be subdivided in monoarthritis or oligoarthritis, including the involvement of one or fewer than five joints, respectively, and polyarthritis, including the involvement of five or more joints.^16^,^23^,^24^

It is important to appreciate that both peripheral and axial arthritis are relatively rare conditions, whereas unspecific or mechanical joint or back pain are very prevalent in the general population as well as in IBD patients, and this large difference in a priori risk should be taken into account when assessing a patient.

### Clinical inflammatory arthritis

If arthritis – either peripheral or axial – is present, this can be diagnosed as IBD-related arthritis, and this syndrome is categorised in the group of SpA – a broad term that covers interrelated inflammatory articular diseases in which axial as well as peripheral joints can be involved.^8^,^25^,^26^,^30^ Of note, arthritis in IBD can of course also be due to sporadic unrelated other forms of inflammatory arthritis such as rheumatoid arthritis or gout.

In general, around 8% of IBD patients (predominantly CD patients) are diagnosed with SpA, of whom 2–4% represent axial SpA.^7^,^11^,^13^,^16^,^21^,^25^ Axial SpA includes inflammation of the SI joints and the subtype AS.^7^,^13^,^15^,^16^,^23^,^25^ The latter manifests itself in various ways, including spondylodiscitis and sacroiliitis and sometimes with concomitant peripheral SpA features.^22^,^23^,^25^,^26^ Rheumatologists use the Assessment of Spondyloarthritis International Society (ASAS) criteria to classify both axial and peripheral SpA (see Supplemental Figure S1 adapted from Rudwaleit et al., 2011). However, we will not discuss these criteria, as their utility is limited for the IBD population.^1^,^3^,^22^,^28^,^29^

In peripheral SpA, arthritis can be present in all joints other than the spinal/axial joints, and also includes the rarer entities such as enthesitis and dactylitis.^3^,^7^,^11^,^16^,^23^,^25^,^31^ In enthesitis, the inflammation affects the insertion of a tendon to the bone, most frequently seen in the Achilles tendon or plantar fascia at the calcaneus.^16^,^22^,^25^ In dactylitis, extended inflammation of the entire finger or toe results in a typical sausage-like appearance.^25^,^26^ Peripheral SpA occurs in up to 25% in IBD patients and more often in CD patients than in UC patients (10–26% and 5–14%, respectively).^5^,^7^,^11^,^16^,^21^,^26^

### Non-inflammatory joint complaints

Non-inflammatory joint complaints – also called unspecific joint pain, arthralgia or arthropathy – can be defined as joint pain in the absence of clinical inflammatory arthritis. It is often seen as a diagnosis of exclusion and includes the ‘regular’ most common types of joint pain such as osteoarthritis (the term for degenerative joint disorder) and other mechanical causes of joint pain, frequently reported in the elderly population or in patients with physically demanding jobs.^16^,^21^,^26^ It is important to realise that the initiation of certain drugs (e.g. anti-tumour necrosis factor (TNF) agents, vedolizumab (VDZ) and ustekinumab (UST)) or withdrawal of certain drugs (e.g. corticosteroids) can also trigger joint pain.^8^,^14^,^26^,^32–35^

How can we differentiate inflammatory arthritis from non-inflammatory joint complaints?

First of all, when septic arthritis or spondylodiscitis are suspected, urgent referral is important. This is in case of acute-onset back pain or joint swelling in one or a few joints (monoarthritis or oligoarthritis), accompanied by fever and elevated inflammatory parameters.^21^ In less acute scenarios, it can be difficult to make a distinction between non-inflammatory and inflammatory joint disease in IBD, as joint complaints can be seen as a spectrum starting from unspecific joint pain (or arthralgia) without suspected inflammation, via clinically suspected arthralgia with symptoms indicating (imminent) arthritis, eventually to a clearly observed and diagnosed clinical inflammatory arthritis (SpA). Because of these difficulties, referral to a rheumatologist is required to rule out or confirm the presence of inflammation and, if indicated, to perform further investigations to establish a clear diagnosis.^21^,^22^,^28^

Physical examination can reveal signs of inflammation, including visible redness, palpable warmth and tender joints or tendon insertion areas and the absence of the normal joint cleft during palpation.^21^ In contrast to other causes of peripheral arthritis such as rheumatoid arthritis, peripheral SpA in IBD often displays an asymmetrical distribution.^21–23^,^26^ Subtle signs of inflammation can be difficult to recognise, and therefore physical examination of the joints is often performed by a rheumatologist. Laboratory results are often not sensitive and specific enough, and it is advised to consult a rheumatologist before ordering HLA-B27 or specific rheumatological antibody tests. US can be used to detect effusion of the synovial fluid in enthesitis or arthritis, but this technique is not performed on regular basis, as its interpretation can be difficult.^26^,^36^

**Figure 1.**
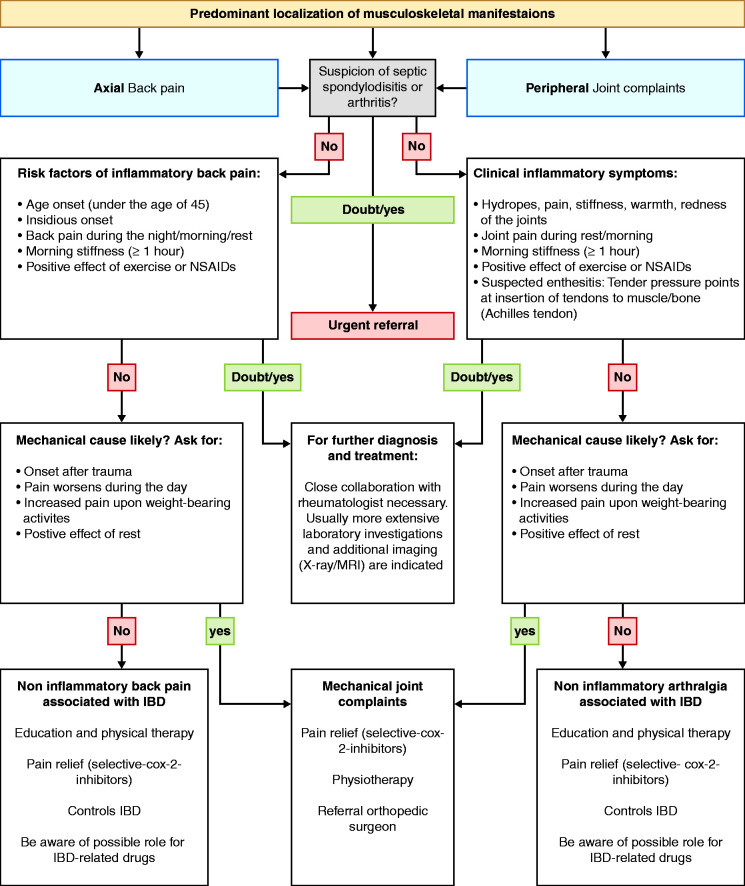
Flow chart for gastroenterologists with suggested approach for the management of IBD patients with joint complaints. IBD: inflammatory bowel disease; NSAIDs: non-steroidal anti-inflammatory drugs; X-ray: radiographic imaging technique; MRI: magnetic resonance imaging technique; COX: cyclooxygenase.

Of note, for identification of SpA in uveitis patients or psoriasis patients, validated diagnostic tools are available for triage, but for IBD, these have not been developed yet. Overall, for establishing a diagnosis in IBD patients with joint complaints, physical examination and imaging techniques are most valuable when performed by a rheumatologist. A stepwise referral strategy for IBD patients with unexplained joint complaints is still missing. Therefore, a suggested clinical algorithm has been designed to help gastroenterologists in this decision-making process (Figure 1).

### How to approach joint symptoms in IBD patients

In contrast to non-inflammatory joint complaints, it is important to recognise and treat axial and peripheral SpA at an early stage in order to prevent pain, additional symptoms, function loss and inflammation.^2^,^22^,^26^,^31^ It is advised to refer an IBD patient with chronic low-back pain (for at least three months) with an insidious onset before the age of 45 years, morning stiffness (persistent for about an hour) with good effect of exercise and/or with peripheral joint pain or swelling or with the presence of dactylitis or enthesitis to a rheumatologist in order to diagnose and treat SpA.^8^,^16^,^21^,^28^,^37^ As it is difficult to distinguish peripheral SpA in IBD from other underlying causes of peripheral arthritis, it is advised to consult a rheumatologist in case of arthritis or clinically suspect arthralgia, in particular with the concomitantly presence of psoriasis, anterior uveitis or a positive familiar history of SpA.^21^,^28^,^37^ As both inflammatory and non-inflammatory types of joint complaints have a great impact on the QoL of IBD patients, they all require early recognition and an adequate treatment approach.^3^,^4^ Head-to-head strategy studies about the therapeutic approach are still missing, and the current literature often takes arthralgia together with arthritis without differentiating the subdivisions used below.^38^ We as research group previously reported on real-live registered data about IBD patients using newer therapies, including UST and VDZ in IBD patients, and similar to other studies, both seem effective to treat IBD patients with arthralgia/arthritis.^21^,^32^ Conflicting evidence about VDZ and UST causing (paradoxical) arthralgia^14^,^33^,^34^ could be explained by the fact that during the induction phase of VDZ and UST, steroids are often simultaneously tapered, causing an increase in joint complaints. Randomised controlled trials (RCTs) are needed to clarify this topic.

### Axial SpA

Sulfasalazine, methotrexate (MTX) and thiopurines do not seem to be effective in treating axial SpA.^8^,^21^,^24^,^26^,^39^ However, mesalamine should be maintained for UC patients in order to maintain remission and for its possible role in the prevention of colorectal cancer. Thiopurines can also be maintained as a combined therapeutic option for immunomodulator naïve CD patients.^24^

#### Active IBD

The first choice to treat axial SpA in active IBD is anti-TNF agents.^8^,^12^,^24^,^26^ In case of non-response, the dose can be increased, the interval shortened or the anti-TNF agent can be switched to another.^24^ In case axial SpA is in remission, continuing with adalimumab is advised, as the risk of recurrence after treatment cessation seems high. However, ongoing research is conflicting, and the evidence is inconclusive at the moment.^24^,^40^

#### IBD in remission

When IBD is in stable remission, short-term use (less than two weeks) of non-steroidal anti-inflammatory drugs (NSAIDs), including selective cyclooxygenase (COX)-2 inhibitors, is an option, but there should be a low threshold to start anti-TNF agents in order to prevent complications of ongoing axial SpA, especially if anti-TNF agents previously had a positive effect on the intestinal disease activity.^8^,^12^,^24^,^26^

### Peripheral SpA

#### Active IBD

Effective therapies for oligoarthritis and polyarthritis are local steroid injection, a low dose of systemic steroids or sulfasalazine (2 g/day or 4 g/day, respectively), whereas the effectiveness of the latter remains inconclusive.^8^,^24^,^26^,^35^,^39^ In IBD, the preferred therapy for both intestinal and peripheral joint inflammation could be sulfasalazine in mild IBD (whereas topical mesalamine should be maintained in distal active UC) and systemic steroids, immunomodulators, anti-TNF agents (infliximab and adalimumab), ustekinumab (interleukin (IL)-12/23-inhibitor) or tofacitinib (Janus kinase inhibitor) in moderate to severe IBD.^12^,^24^,^26^,^35^,^38^,^41^

#### IBD in remission

Similar to axial SpA, for both oligoarthritis and polyarthritis, short-term use of selective COX-2 inhibitors is accepted in inactive IBD, preferably used to bridge local injections of steroids in oligoarthritis and oral sulfasalazine therapy (2–3 g/day) for polyarthritis.^24^,^26^,^35^ In case of non-response, anti-TNF agents are a safe second therapeutic option.^24^,^35^,^38^

### Non-inflammatory arthralgia

For non-inflammatory joint complaints, treatment options are generally limited to physical therapy and/or a stepwise approach of analgesics, starting with acetaminophen (though not effective in osteoarthritis) and adding COX-2 inhibitors (such as etoricoxib or celecoxib) as a second step.^21^,^26^ In case of osteoarthritis, in some cases, referral to an orthopaedic surgeon is indicated and helpful.^21^

In Table 2, an overview of all the above-mentioned therapeutic options for musculoskeletal manifestations in IBD patients are summarised.

**Table 2.** Overview of first-, second- and third-line therapy of EIM in patients with IBD.

Therapeutic approach EIMs	First line therapy	Second line therapy	Third line therapy
Musculoskeletal			
Axial SpA	COX-2 inhibitorsAnti-TNF agents	Anti-TNF agents	
Peripheral SpA	Systemic steroidsSulfasalazineMethotrexate Local steroid injection COX-2 inhibitors	Anti-TNF agents	IL-12/-23 inhibitorsJAK inhibitors
Non-inflammatory arthralgia in IBD	Physical therapyCOX-2 inhibitors	None	None
Cutaneous			
Erythema nodosum	Control underlying IBDCOX-2 inhibitors	Short course (1–2 weeks) of systemic steroids Hydroxychloroquine	
Pyoderma gangrenosum	Wound care Topical therapy (steroids or tacrolimus)	Systemic steroids Calcineurin inhibitor: Oral cyclosporine or tacrolimus Azathioprine or methotrexate	Anti-TNF agents
Hidradenitis suppurativa	Topical antibiotics (clindamycin) Oral tetracycline	Surgical incision and drainage Combination therapy of multiple antibioticsAcitretin	Anti-TNF agents
Psoriasis	Topical steroids, derivatives of vitamin D, tacrolimus, phototherapy, photochemotherapy	Conventional systemic therapies: MethotrexateCyclosporineAcitretinFumaric acid esters	Anti-TNF agentsIL-12/23 inhibitorsIL-23 inhibitors
Ocular			
Episcleritis	Self-limiting	Topical steroids	
Scleritis	Dexamethasone eye drops	Systemic steroids	
Anterior uveitis	Topical/ systemic steroids	Anti-TNF agents	

In all cases, active intestinal disease activity, if present, should have priority in the management of EIMs. Suggested treatments are traded towards treatment of intestine symptoms and EIMs.COX: cyclooxygenase, EIMs: extraintestinal manifestations, IBD: inflammatory bowel disease, IL: interleukin, JAK: janus kinase, PG: pyoderma gangrenosum, SpA: spondylarthritis, TNF: tumor necrosis factor.

### Are NSAIDs safe for IBD patients?

The use of NSAIDs in IBD, especially the ones with relative high selectivity for COX-1 inhibition, is usually discouraged because of the possible risk of an exacerbation of the underlying IBD.^8^,^26^,^35^ Generally, a selective COX-2 inhibitor is preferred as a safer alternative.^8^,^26^,^42^ However, evidence about the effect of conventional (COX-1) inhibitors on intestinal disease activity is conflicting, as some studies have found early clinical relapses right after the start of NSAIDs,^43^ whereas other studies have not found a clear association between NSAIDs and the risk of intestinal exacerbation.^44^ It is generally believed that COX-2 inhibitors have a reduced likelihood of inducing intestinal flares compared to COX-1 inhibitors. However, these somewhat older studies are based on less used COX-2 inhibitors, or they compared COX-2 inhibitors to placebo rather than COX-1 inhibitors.^42^,^43^,^45^ In short, evidence on this topic is inconsistent and based on older and heterogenic studies. So, further research will be necessary to find out more about the safe use of different types of NSAIDs. Awaiting further research on this topic, the use of selective COX-2 inhibitors is the preferred strategy, while short-term treatment with relative COX-1-selective NSAIDs might be a safe alternative if the underlying IBD is in remission.^8^,^21^,^26^,^30^,^35^,^44^,^45^

## Key points: inflammatory joint complaints in IBD

- Rule out septic arthritis first and use history, physical examination, laboratory results and imaging techniques to differentiate.
- Strive for a multidisciplinary treatment approach together with a rheumatologist and consider drugs that simultaneously treat IBD and joints:
- Axial SpA and active IBD: anti-TNF agents are the first choice;
- Axial SpA and IBD in remission: short-term use of COX-2 inhibitors or anti-TNF agents in non-responders;
- Peripheral SpA and active IBD: in mild IBD, local steroidal injection, sulfalasazine, low-dose steroids; in moderate to severe IBD, anti-TNF agents;
- SpA and quiescent IBD: short-term use of selective COX-2 inhibitors or sulfalasazine, or anti-TNF agents in non-responders.

## Key points: non-inflammatory joint complaints in IBD

- Diagnosis of exclusion, which can be IBD related or can have various alternative underlying causes (degenerative, mechanic, therapeutic side effects or withdrawal induced).
- Treatment: physical therapy or COX-2 inhibitors (which are preferred over COX-1 inhibitors, particularly for long-term use).

## Clinical case continued 2

After a week, she develops more red-coloured painful lesions located on the anterior surface of the right tibia. Based on the typical clinical features, she is diagnosed with EN, and a short course of low-dose prednisone is started. Within three to four weeks, the nodules have completely resolved without scar formation. The joint pain in her ankles and knees simultaneously disappeared.

## Cutaneous manifestations

### How to differentiate cutaneous manifestations in IBD patients

In general, about 20% of IBD patients at some time point report concomitant skin disorders. Given the extensive differential aetiology for skin lesions, here we discuss four diagnoses with specific clinical relevance in IBD patients. With a prevalence ranging from 1% to 15% in IBD patients, EN is the most common, particularly in CD patients (7–15% compared to 2.8–10% in UC patients).^5^,^7^,^11^,^13^,^15^,^17^,^19^,^46^ PG is a rare EIM, occurring in only 0.8–5% of IBD patients, and in contrast to other EIMs, PG is more common in UC (0.9–8%) than in CD (0.7–3.5%) and has a potentially severe impact on QoL.^5^,^7^,^11^,^13^,^15^,^17^,^19^,^47^ Another important skin disorder to take into account is HS (also called acne inversa), generally considered as a distinctive (or IBD-associated) disease, but with a prevalence of up to 23% in IBD patients (0.4–15% in CD and 0.1–6.1% in UC) compared to 0.1–4% in the general population.^7^,^48–50^ Psoriasis has been seen as a disease associated with IBD and occurs in 2.7–8.3% of IBD patients, with a higher prevalence in CD (2.8–3.3%) than in UC (2.1–2.9%).^7^,^11^,^15^,^51^

**Figure 2.**
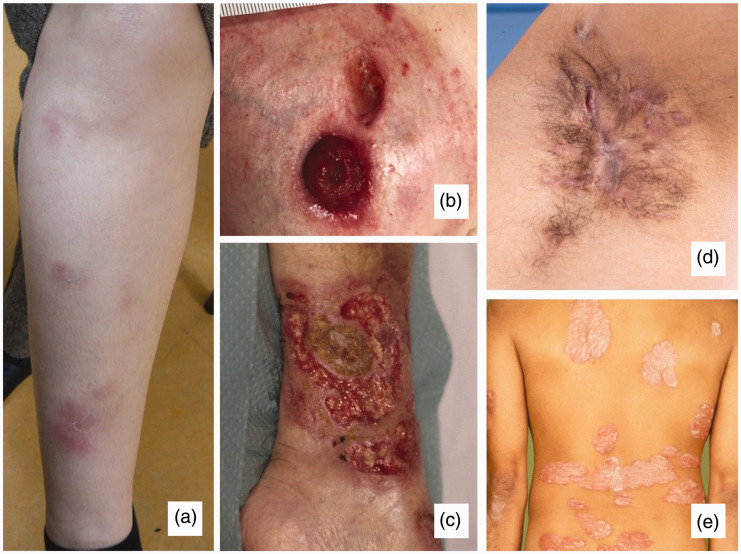
Cutaneous manifestations: erythema nodosum located on the anterior shin (a), peristomal pyoderma gangrenosum (b), pyoderma gangrenosum located at the lower leg (c), axillary hidradenitis suppurativa (d) and psoriasis vulgaris (e). Adapted from personal archive ((a) and (b)) and www.huidziekten.nl ((c), (d) and (e)).

EN is characterised by painful, slightly raised, subcutaneous red-violet nodules 1–5 cm in diameter located on the extensor surfaces of the lower extremities (anterior tibia; Figure 2).^8^ EN can be triggered by a broad range of underlying conditions, such as other inflammatory diseases (e.g. sarcoidosis), infections (*Streptococcus*, *Tuberculosis*), malignancies, drugs (sulphonamides, contraceptive pills) or pregnancy.^2^,^9^ In EN, skin biopsies are seldom required because of the very typical clinical picture of the lesions.^8^

PG has different subtypes, but in the most common classic form, which is the ulcerative variant, PG starts with a (painful) erythematous nodule or plaque, sometimes with small erythematous sterile pustules. Consecutively necrotic ulcerative areas with violaceous irregular oedematous edges develop, which can rapidly extend to surrounding areas.^8^ The ulcers contain sterile purulent material, vary in size from 2 to 20 cm in diameter and can be the source of the development of superinfections or sepsis.^8^,^47^ Most common localisations of PG are the lower legs (pretibial) and peristomal areas (Figure 2) in up to 80% and 18% of cases, respectively, but PG can appear anywhere on the body surface.^47^,^52^ PG as an ulcerative disorder has a broad differential diagnosis, which can be subdivided into vascular (venous, arterial, occlusive or vasculitis) diseases, haematological diseases (polycythemia vera), malignancies, infections and drug-induced tissue injury.^52^,^53^ In PG, infectious causes can be ruled out by skin swabs of the ulcerative lesions. Biopsies often show an unspecific neutrophilic infiltration and necrosis. Signs of vasculitis can also be seen. Biopsies are preferred over skin swabs to rule out other kinds of underlying diseases, including malignancy.^2^,^8^,^53–55^ This has to be done with caution, as a typical phenomenon called pathergy can occur after a preceding trauma and can often contribute to the expansion of PG lesions.^47^,^52^,^54^

In HS, the diagnosis is often established by lesion morphology, location and lesion progression rather than skin biopsies.^56^ HS is characterised by recurrent formation of painful inflamed skin lesions, developing abscesses and interconnected sinus tracts mainly at inverse body regions, such as the inguinal, axillary and peri-anal area (Figure 2).^10^,^48^,^49^,^56^ In HS, the diagnostic process can be delayed because HS sometimes resembles a simple skin infection or carbuncles/furuncles in an early stage. Besides, HS in peri-anal regions can sometimes be hard to distinguish from peri-anal fistulas in Crohn’s disease.^48^,^50^,^56^

Of the different forms of psoriasis, psoriasis vulgaris or the chronic plaque psoriasis is the most common subtype (Figure 2).^57^,^58^ Psoriasis vulgaris is characterised by the presence of clearly defined monomorphic erythematous plaques with silver-coloured gill-like scales.^57^,^58^ Psoriasis is commonly localised at extensor areas of the elbows, knees, scalp, peri-umbilical and peri-anal areas.^57^ Flexural (skin-fold) areas, nails, scalp and joints (psoriatic arthritis) can be involved, the latter in up to 30% of patients with moderate to severe psoriasis.^57^ Other subtypes of psoriasis can manifest as sterile pustules instead of plaques.^57^ Psoriasis can be triggered by mild trauma, systemic drugs or infectious diseases (e.g. human immunodeficiency virus).^57^ Specific scoring systems for disease activity are used, such as the Psoriasis Area and Severity Index or the Physician Global Assessment.^57^,^59^ Other cutaneous diseases could give difficulties in establishing a diagnosis, including tinea pedis or corporis, seborrheic dermatitis or eczema.^57^ An important form of psoriasis is paradoxical psoriasis, which can be induced by treatment with anti-TNF agents, especially infliximab and adalimumab.^38^,^51^,^57^,^58^ Paradoxical psoriasis is clinically very similar to classic psoriasis, but its inflammatory pathway is different and is dominated by interferon type 1.^58^ Treatment is difficult, and withdrawal of the drug that caused this type is often necessary.^58^

### How to approach cutaneous manifestations in IBD patients

In IBD patients with EN, the most likely trigger is intestinal disease activity of the underlying IBD, and adequate treatment of the IBD will frequently lead to resolution of EN without scar formation.^7^,^12^,^38^ In case of refractory EN or when in doubt, referral to a dermatologist can be helpful to establish a definite diagnosis. In severe cases where lesions can be very painful, a short course of oral corticosteroids (0.5–1 mg/kg/day for one or two weeks) usually leads to rapid resolution of EN.^8^,^9^ In collaboration with a dermatologist, hydroxychloroquine can be a second-line therapy.

If PG is suspected, the diagnosis and treatment should take place in close collaboration with a dermatologist, as PG has an unpredictable and damaging disease course due to pain, frequent recurrences, scarring, secondary infections and even sepsis.^9^,^47^ Delay in the recognition and treatment can lead to progression of the lesion and subsequent complications.^53^,^54^ In case intestinal disease activity is present, treating the underlying IBD often results in improvement of PG.^5^,^38^,^47^ Important in the treatment of PG are wound care, pain management and exclusion of skin infections before initiating immunosuppressants. Surgical interventions (excision) should be avoided if possible, as traumas may worsen PG lesions.^9^ In mild cases, topical therapy can be used such as corticosteroids or topical tacrolimus.^9^,^47^ In moderate to severe cases, systemic (oral) corticosteroids such as prednisolone (0.5–2 mg/kg per day), calcineurin inhibitors such as oral tacrolimus (0.3 mg/kg per day) or cyclosporine (4–5 mg/kg per day) can be required.^9^,^47^ To prevent long-standing use of corticosteroids, azathioprine and MTX are good alternatives as maintenance strategy for both PG and IBD in order to prevent recurrence. Anti-TNF agents – infliximab and adalimumab in particular – are very effective treatment options in case of delayed response to corticosteroids.^38^,^47^,^55^ Aggressive and prolonged therapy is required until the PG lesions are completely healed.^47^

In general, factors associated with cutaneous manifestations are smoking and obesity, and discussing these lifestyle factors is particularly important as a first step in the treatment of patients with HS.^19^,^49^,^50^ HS is notorious for its debilitating disease course and difficulties in treatment. Close collaboration with dermatologists and surgeons is advised to treat HS aggressively in order to prevent long-term inflammation, fibrosis and scarring.^48^,^56^ To treat the inflammation, the first choice in mild cases is topical clindamycin.^56^ Prolonged treatment with oral tetracycline, combination therapies consisting of multiple antibiotics or treatment with acitretin can be considered in moderate to severe HS.^48^,^56^ For single nodules, oral intra-lesional steroids could be effective.^48^,^56^ RCTs have shown that intravenous infliximab and subcutaneous adalimumab are effective therapies for moderate to severe HS.^60^,^61^ HS in IBD patients often follows a more severe disease course than in patients without IBD, and in severe cases, early and surgical wide incision and drainage reduces the risk of recurrence from about 38.5% to 8%.^48^,^56^

In IBD patients with psoriasis or concomitant psoriatic arthritis, a multidisciplinary approach is advised together with the dermatologist and/or rheumatologist. Mild psoriasis can be treated with topical therapy, including corticosteroids, derivatives of vitamin D and calcineurin inhibitors (tacrolimus) for sites with persistent disease activity.^57^ In moderate to severe psoriasis, phototherapy or photochemotherapy, including narrow-band ultraviolet B or psoralen plus ultraviolet A, respectively, are effective but often not enduring.^57^ Conventional systemic treatment includes methotrexate, cyclosporine, acitretin and fumaric acid esters.^57^,^59^ Infliximab and adalimumab are effective in treating both IBD and psoriasis, whereas other biologicals such as IL-17 inhibitors can affect the intestinal disease activity.^57^,^59^ New biologicals, such as IL-23 antagonists, are registered for psoriasis and may also be effective treatment for patients with IBD and psoriasis.^62^

Table 2 gives an overview of therapeutic approaches for cutaneous manifestations in IBD patients.

### Summary

## Key points: erythema nodosum

- Clinical diagnosis with red-violet nodules typically located on the shins.
- In IBD: EN mirrors the intestinal disease activity.
- Treatment: self-limiting as soon as underlying IBD is adequately treated. Consider corticosteroids for rapid resolution of symptoms.

## Key points: pyoderma gangrenosum

- Diagnosis of exclusion, broad differential diagnosis.
- Treatment: mild cases with topical therapy, moderate to severe cases with systemic anti-inflammatory therapy, including corticosteroids, calcineurin inhibitors or anti-TNF agents.

## Key points: hidradenitis suppurativa

- Unpredictable debilitating disease course with reduced QoL.
- Treatment: antibiotics, anti-TNF agents, surgery (incision and drainage).

## Key points: psoriasis

- Associated skin disease of IBD; can also manifest in the nails and joints (psoriatic arthritis).
- Multidisciplinary approach: topical therapy calcineurin inhibitors, corticosteroids or photo(chemo)therapy or systemic therapy (anti-TNF agents or ustekinumab).

## Clinical case continued 3

A few years later, the same patient calls the outpatient clinic because of a slightly painful eye combined with photophobia and pain. There is no redness of the eye. Her UC is in clinical remission under azathioprine and mesalamine.

After immediate referral to an ophthalmologist, the patient is diagnosed with iridocyclitis, which is successfully treated with intraocular corticosteroids.

### How to differentiate reactive ocular manifestations in IBD

**Figure 3.**
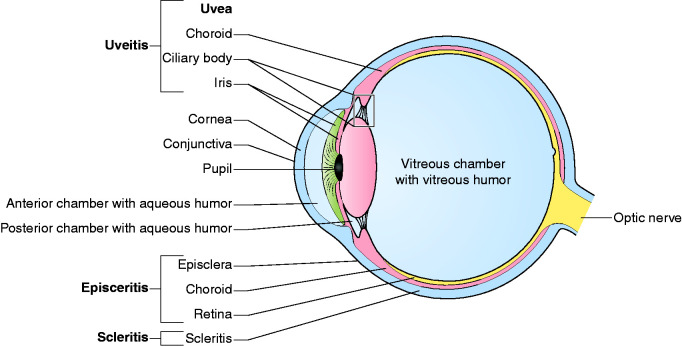
Anatomical overview of the eye and ocular manifestations.

The most common ocular EIMs in IBD are episcleritis and anterior uveitis, with prevalence rates of 2–4% and 1.7–5%, respectively, whereas anterior uveitis seems more common in CD (1.5–11%) than in UC (0.7–10.5%).^7^,^11–13^,^15^,^63^,^64^ With a prevalence of <1%, scleritis and other types of uveitis, including intermediate and posterior uveitis, are less frequent.^1^,^15^,^65^ For an overview of the anatomy of the eye, see Figure 3.

In episcleritis, a benign and often recurrent inflammation of the episcleral causes acute redness, irritation, tearing and mild-to-moderate discomfort in one or both eyes.^2^,^66^,^67^ Episcleritis can be discriminated from scleritis or uveitis by the absence of visual impairment or ocular pain.^8^,^66^ Episcleritis can sometimes be difficult to distinguish from conjunctivitis, which is a benign and often self-limiting disorder, as in both conditions hyperaemia is usually present.^15^,^20^,^63^,^67^

Scleritis is characterised by deeply inflamed sclerae, causing scleral oedema.^66^ Typical features are the presence of severe ocular ache radiating to the scalp and face that worsens at night and can cause visual loss.^8^,^67^ In the most common type of anterior scleritis, hyperaemia is visible, which is often not the case in posterior scleritis.^67^

In uveitis, inflammation of the middle layer of the eye (the uvea) can affect the iris, ciliary body and/or choroid (Figure 3).^68^ This may lead to one of four different types of uveitis: anterior, intermediate, posterior or pan-uveitis. Anterior uveitis is the most common type in IBD.^1^,^8^,^63^,^68^ The symptoms depend on the localisation of inflammation, but it is mostly characterised by the presence of ocular pain, blurred vision, photophobia and headaches.^2,8,63,67^ A typical feature of anterior uveitis is the presence of a hypopyon which is formed by accumulation of inflammatory cells into the anterior eye chamber, causing a visible pocket with pus.^2^,^68^

### How to approach ocular manifestations in IBD patients

In case of impaired vision and ocular pain rather than discomfort, there should be a strong suspicion of scleritis or uveitis, and immediate referral to an ophthalmologist is necessary, as both scleritis and uveitis can result in permanent visual loss if left untreated.^1^,^8^,^63^,^66^,^67^

As episcleritis often parallels the intestinal disease activity, it is important to control the underlying IBD.^7^ Episcleritis is mostly self-limiting, and topical corticosteroids are rarely necessary.^1^,^2^,^67^ A wait-and-see approach is advised. In case of doubt about the diagnosis or in case of the development of new and alarming symptoms, referral to an ophthalmologist is indicated.

Treatment of scleritis and uveitis is usually carried out by an ophthalmologist. In case of scleritis, topical anti-inflammatory agents such as dexamethasone eye drops or systemic corticosteroids are necessary and contribute to a good prognosis.^2^,^65^,^66^ In case of uveitis, in mild cases, topical corticosteroids may be sufficient, but regularly (and in more severe cases), systemic corticosteroids or anti-TNF agents are required.^8^,^38^,^64^

Table 2 gives an overview of the above-mentioned therapies for ocular manifestations in IBD patients.

## Key points: episcleritis

- Common ocular manifestation in IBD, without ocular pain or vision impairment.
- In IBD: related to intestinal disease activity.
- Treatment: self-limiting.

## Key points: scleritis

- Rare ocular manifestation in IBD characterised by severe radiating ocular pain and impaired vision.
- Management: immediate referral to an ophthalmologist to prevent permanent loss of vision; often treated with local or systemic corticosteroids.

## Key points: uveitis

- Of the four types of uveitis, anterior uveitis is the most common, characterised by ocular pain, photophobia and blurred vision.
- Treatment: urgent referral to an ophthalmologist; often treated with systemic corticosteroids, biologicals or anti-TNF agents.

### Future perspectives

Evidence-based knowledge on the pathogenesis of EIMs in IBD is lacking, and two theories cover most of the current theories.^1^ The first theory is that EIMs can be seen as disseminated immune-affecting extra-intestinal localisations, caused by, for example, microbial antigenic cross-reactivity or translocation.^1^ This theory would imply that faecal transplantation or the use of pre- or probiotics are potential targets for the treatment of EIMs. Research is ongoing, and the evidence is currently inconclusive. A second theory is that the inflammatory events causing EIMs and those causing IBD are considered independent inflammatory entities provoked by similar environmental or genetic factors in susceptible patients.^1^,^30^ From this point of view, the differences between reactive and associated EIMs in IBD can be explained. However, with the absence of stringent definitions of musculoskeletal manifestations and limited literature about ocular manifestations and EN in IBD, more research is required to understand which pathways are involved and to improve the treatment approach. The latter is the biggest issue for gastroenterologists during their outpatient visits. Whereas Varkas et al. designed a useful stepwise treatment approach for musculoskeletal manifestations in IBD, golden standards for the treatment of EIMs in IBD and therapeutic options are still warranted, and the questions (shown in Supplemental Figure S2) remain unanswered.^21^

## Conclusion

Almost half of IBD patients report EIMs of which musculoskeletal manifestations are the most common, followed by cutaneous and ocular manifestations. When coming across (suspected) EIMs during the treatment or follow-up of an IBD patient, close collaboration with rheumatologists, dermatologists and ophthalmologists is advised in order to prevent diagnostic delays and irreversible damage. A research agenda aimed at further elucidating the pathogenesis of EIM and to establish evidence-based therapeutic approaches is crucial to improve QoL for IBD patients.

## Supplemental Material

sj-pdf-1-ueg-10.1177_2050640620958902 - Supplemental material for Clinical management of the most common extra-intestinal manifestations in patients with inflammatory bowel disease focused on the joints, skin and eyes Click here for additional data file. Supplemental material, sj-pdf-1-ueg-10.1177_2050640620958902 for Clinical management of the most common extra-intestinal manifestations in patients with inflammatory bowel disease focused on the joints, skin and eyes by Fenna M Jansen, Stephan R Vavricka, Alfons A den Broeder, Elke MGJ de Jong, Frank Hoentjen and Willemijn A van Dop in United European Gastroenterology Journal
