# Supplementary material for: Clinical management of the most common extra-intestinal manifestations in patients with inflammatory bowel disease focused on the joints, skin and eyes
Source: United European Gastroenterol J. 2020 Sep 14;8(9):1031–44. doi: 10.1177/2050640620958902 (PMC7724540; doi:10.1177/2050640620958902)
Supplement: sj-pdf-1-ueg-10.1177_2050640620958902 - Supplemental material for Clinical management of the most common extra-intestinal manifestations in patients with inflammatory bowel disease focused on the joints, skin and eyes [file sj-pdf-1-ueg-10.1177_2050640620958902.pdf]

Supplementary File 1: **Axial and Peripheral ASA-criteria**

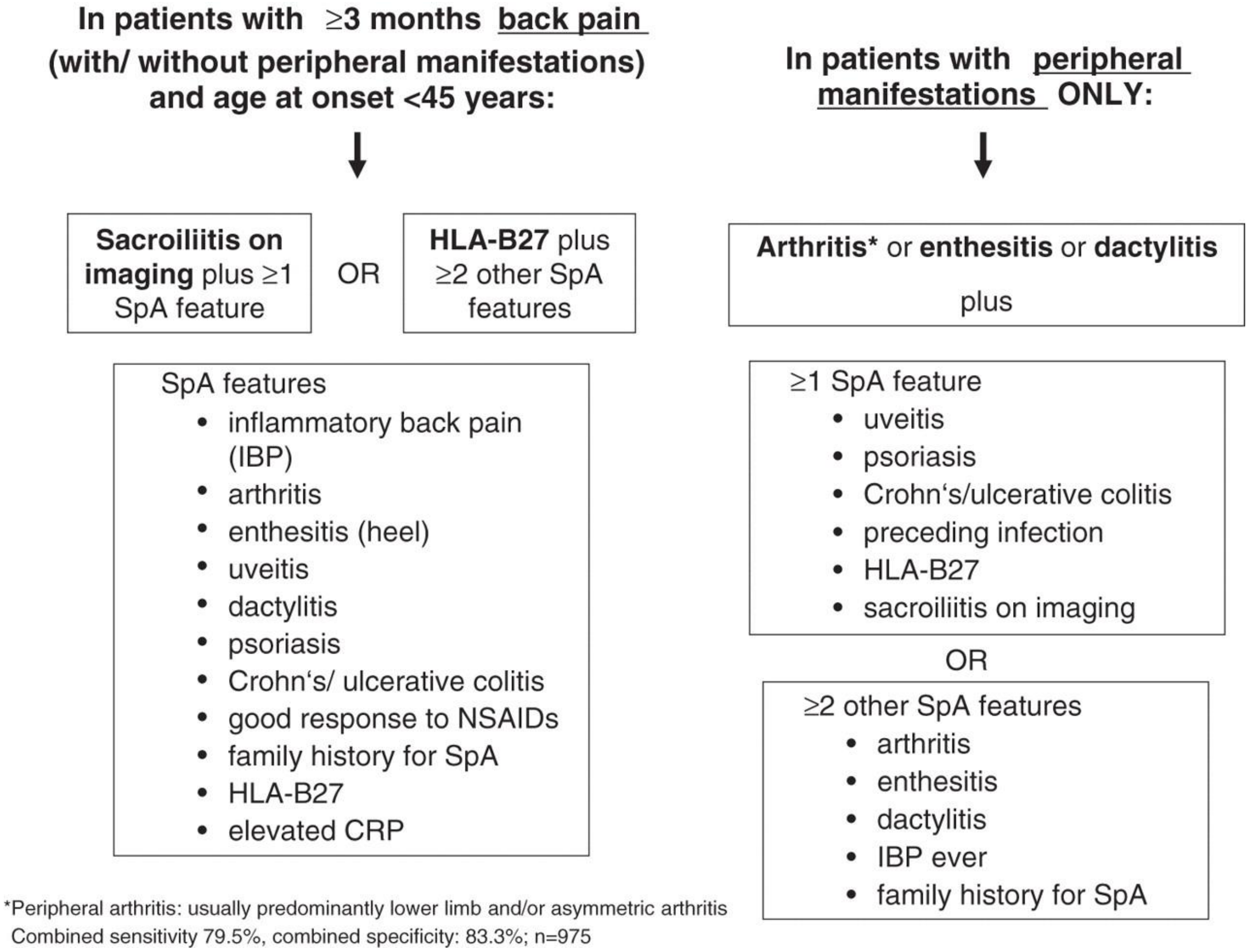

Supplementray Figure 2

Future perspectives	
**Pathogenesis**	Which (non-)inflammatory pathways result in EIM
	What is the role of the microbiome in the pathogenesis of IBD
	What is the role of IBD disease activity in the burden of EIM
**Treatment**	How can we quantify severity and symptoms of EIM
	What therapeutic intervention is effective for a specific EIM
	What sequential strategies achieve optimal outcomes in EIM
